# Early mutation bursts in colorectal tumors

**DOI:** 10.1371/journal.pone.0172516

**Published:** 2017-03-03

**Authors:** Junsong Zhao, Matthew P. Salomon, Darryl Shibata, Christina Curtis, Kimberly Siegmund, Paul Marjoram

**Affiliations:** 1 Department of Molecular and Computational Biology, University of Southern California, Los Angeles, California, United States of America; 2 Department of Molecular Oncology, John Wayne Cancer Institute at Providence Saint John’s Health Center, Santa Monica, California, United States of America; 3 Department of Pathology, Keck School of Medicine, University of Southern California, Los Angeles, California, United States of America; 4 Department of Medicine (Oncology) and Genetics, School of Medicine, Stanford University, Stanford, California, United States of America; 5 Department of Preventive Medicine, Keck School of Medicine, University of Southern California, Los Angeles, California, United States of America; National Cancer Institute, UNITED STATES

## Abstract

Tumor growth is an evolutionary process involving accumulation of mutations, copy number alterations, and cancer stem cell (CSC) division and differentiation. As direct observation of this process is impossible, inference regarding when mutations occur and how stem cells divide is difficult. However, this ancestral information is encoded within the tumor itself, in the form of intratumoral heterogeneity of the tumor cell genomes. Here we present a framework that allows simulation of these processes and estimation of mutation rates at the various stages of tumor development and CSC division patterns for single-gland sequencing data from colorectal tumors. We parameterize the mutation rate and the CSC division pattern, and successfully retrieve their posterior distributions based on DNA sequence level data. Our approach exploits Approximate Bayesian Computation (ABC), a method that is becoming widely-used for problems of ancestral inference.

## Introduction

Tumorigenesis is the process by which normal cells transform to ultimately become malignant and experience uncontrolled growth. During this process, numerous genomic and epigenomic events take place. The normal spontaneous DNA mutation rate ranges from 10^−10^ to 10^−9^ per base per cell division due to replication error [1,2], which means the overall mutation rate is between 0.3 and 3 mutations per cell division in the whole genome, and 0.003–0.03 mutations per cell division per exome. In tumors, the point mutation rate is ~5×10^−10^ per base per division, which is within the ranges for normal cells, based on sequencing and microarray results from pooled DNA [3–5]. The accuracy of these estimates relies on reliable detection of mutations and estimation of the number of divisions the tumor has experienced. However, the technology typically used does not detect all mutations since the probability of detection depends upon depth of sequencing and subclone size. Neither do we typically observe the number of generations through which the tumor has passed during its existence.

In this paper we seek to better estimate point mutation rates in tumors, and to then understand how they might vary during the lifetime of the tumor. A recent study has proposed a “big bang” model of colorectal tumor growth, such that after transformation the tumor grows as a single terminal expansion in the absence of stringent selection [6]. Furthermore, it is proposed that most detectable mutational intratumor heterogeneity observed in genomic data originates largely from the first few divisions [6–8]. An open question is whether the mutation rate during those first few divisions was elevated, resulting in a “mutation burst” at the very beginning of the tumor development, or whether the rate was constant during tumor development. Colorectal tumors provide an exceptional advantage when investigating this issue, because of their glandular structure. In this paper we exploit “single-gland” tumor sequencing data for a number of single glands from a tumor. Such data is currently rare, but is expected to become more common. Each gland within the tumor is a relatively pure cell population, and mutations that originate early in the tumors history will typically be present in all cells within a gland (this kind of mutation is therefore called a “fixed” mutation in this manuscript) [8]. It is the prevalence of such fixed mutations, relative to that of the non-fixed mutations that will be informative regarding the existence of an initial mutation burst. Therefore, profiling single glands, and looking for this signal, for example, using exome sequencing, will provide us with unprecedented information regarding the initial mutation burst. On the contrary, traditional bulk tissue sampling that consists of thousands of glands loses this power, since both structural information and the ability to determine that a mutation is fixed within one or more glands is obscured. In this paper we develop the methodology to permit analysis of single-gland data, and present an exemplar analysis of an early example of such data: *new single-gland* exome sequencing data from one tumor, tumor U previously studied by Sottoriva et al., 2015 [6].

Stem cells are undifferentiated cells that reside in multicellular organisms. They are capable of making more stem cells, a process called self-renewal, as well as generating other types of cells, a process known as differentiation. Stem cell division, through which the stem cells self-renew and differentiate, has been extensively studied in simple organisms, for example, C. elegans [9], as well as in higher organisms, such as humans [10]. Two types of stem cell division have been discovered: asymmetric and symmetric. A stem cell that is undergoing asymmetric cell division produces one daughter cell that is itself a stem cell, and one daughter cell that loses stem cell properties and differentiates [11,12]. One of the advantages of this asymmetric division is that it maintains and constrains the cell population while it produces two different cells. This advantage is also its disadvantage under certain circumstances; for example if the stem cell population needs to expand. In contrast, in symmetric cell division, a stem cell divides into two daughter cells that are destined to have identical fate—in other words, both are cells that differentiate or both are stem cells. Symmetric cell division is essential for population expansion of stem cells during the initial stages of embryo development and during wound healing and regeneration [13–15].

Asymmetric cell division has been extensively studied in model systems and many processes are involved in regulating asymmetric division [16–20]. Abnormality in these pathways results in disruption of asymmetric cell division and eventually causes development of cancers [16,21]. Therefore, equilibrium between asymmetric division and symmetric division is crucial to organisms. If the equilibrium is disturbed, abnormal growth will take place and tumors will typically arise. Several studies have shown that some protein markers for asymmetric division are still present during cancer cell division, suggesting that asymmetric cell division is not totally lost in cancers [22].

The partial loss of asymmetric division in cancers suggests a way to study the regenerative ability of a cancer. It may, therefore, be useful to understand to what extent each tumor has lost asymmetric division. Currently, there is no technique that can measure the proportion of stem cells that undergo asymmetric division or predict the probability that a given stem cell will divide asymmetrically. However, these two different division mechanisms change the probability that mutations carried by a cancer stem cell (CSC) will survive into the next generation. For example, if a CSC with a unique mutation undergoes symmetric division and gives birth to two non-stem cells, this mutation will vanish in the stem cell population (and ultimately will vanish from the tumor itself, since differentiated cells have relatively short lifespans). In this paper we propose a simulation-based method that can be used to estimate both the mutation rate and asymmetric division rate of CSCs, and can in addition infer whether a mutation burst occurred in that tumor. More specifically, our tumor simulation framework, which includes the exploitation of next-generation sequencing (NGS) data for single tumor glands, provides researchers with more detailed information on the genomic landscape of a tumor.

## Data, model and methods

### Tumor growth model and DNA mutation embedding

**Fig 1:**
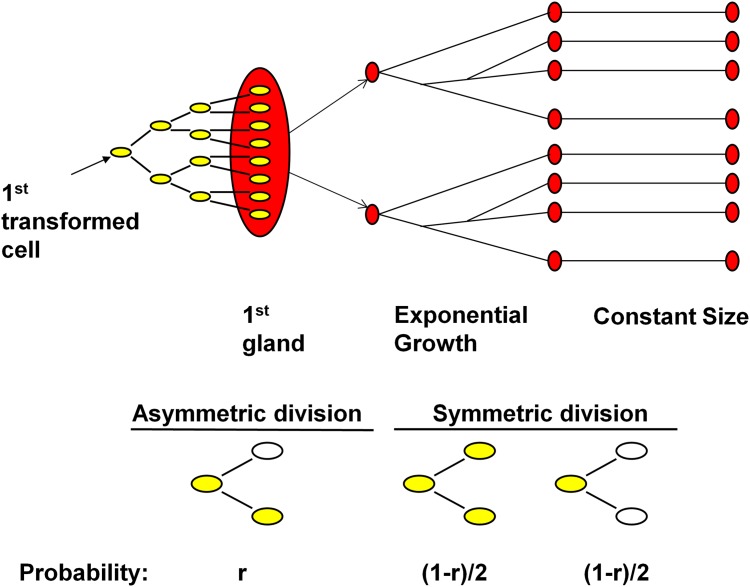
Schematic of tumor growth and the two types of CSC division. (A) The three stages of the growth model: formation of the first gland, exponential growth of gland number, and constant size phase (the length of which is 100 generations). (B). Schematic of cell differentiation process during Constant Size phase. Each yellow oval represents a CSC, while each white oval represents a non-CSC (cells which have limited differentiation capability).

Our analysis models possible scenarios for tumor growth. The model supposes that any given tumor contains a particular number of CSCs. Our simulation model begins with repeated division of the first transformed cell until the number of cells reaches the number of CSCs existing in that tumor. This results in the formation of the first gland (assumed to contain ~10,000 cells). Once the first gland forms, we model a gland fission process. Initially, the tumor experiences an ‘exponential growth’ stage, in which the glands double in number every generation (see Fig 1A), for 19 generations, to ultimately number ~500,000 glands and ~4 billion cells, which is approximately the size of a 4 cm^3^ colon tumor. Then the tumor enters a ‘constant size’ phase, in which the gland fission process stops. During the constant size phase, the cell population in each gland, which consists of both CSCs and non-cancer stem cells (non-CSCs), is maintained by the division of CSCs and the death of non-CSCs. As discussed earlier, a CSC can undergo two types of division: asymmetric and symmetric (see Fig 1B). The probability of asymmetric division, r, is a parameter to be estimated in our model: with probability *r*, a given CSC will undergo asymmetric division, in which only one of the progeny is a CSC. Otherwise, (so with probability 1-*r*), a CSC will undergo symmetric division, in which case, with equal probability, the CSC divides into two CSCs or two non-CSCs. This latter assumption is required in order to maintain a gland of constant size. In other words, any other choice results in a tumor that either consistently grows in size, or decreases in size, depending upon the specific choice of probabilities made, contrary to the standard Gompertzian models of colon tumor growth, in which a period of rapid growth is followed by a long period of constant size. [23,24]. As more data arises, one could test the validity of models in which this latter assumption is relaxed, allowing for some fluctuations in tumor size during this latter ‘constant period’, but such an analysis requires more data than is available currently.

The possibility of DNA mutation is incorporated into each cell division. Since mutation rates are relatively low, we model the number of DNA mutations, n, introduced into each daughter cell according to a Poisson distribution, the mean of which is referred to as the mutation rate. Since we are interested in asking whether there is a mutation burst at the early stage of tumor growth, we parameterize the DNA mutation rate separately before (mutation rate *α*) and after (mutation rate *β*) the first gland formation. Our parameters are summarized in Table 1. Since the focus of this study is the possible existence of an early mutation burst, and the details of stem cell division, in our simulation study NCSC and T3 are set to ‘typical constant’ values (see Table 1) so that we can keep the dimensionality of the simulation study manageable. The prior distribution of *α* and *β* are set to cover a range wider than the mutation rates, 0.015–0.15 mutations per cell division per exome that are typically reported in the literature.

**Table 1:** Parameters in our model.

Parameter	Range of Possible Values and Prior Distribution
DNA point mutation rate before the first gland formation (*α*)	Unif(0,5)
DNA point mutation rate after the first gland formation (*β*)	Unif(0,1)
Number of Cancer Stem Cells (NCSC)	32
Probability of asymmetric division (*r*)	Unif(0.5,1)
Number of generations in constant size phase (T3)	100

### Statistical methods

#### Approximate bayesian computation

Our goal here is to find the posterior distribution of tumor growth parameters, in general denoted by θ = (θ_1_, θ_2_,…, θ_L_), based on data, D, that were observed experimentally. This is expressed as:

$$ƒ\left(\theta |\text{D}\right)=\frac{ƒ\left(\text{D}|\theta \right)\pi \left(\theta \right)}{ƒ\left(\text{D}\right)}.$$ (1)

In our context the likelihood term ƒ(D|θ) is intractable and not available in closed form. Therefore we replace the likelihood calculation with an acceptance-rejection simulation step that accepts parameter values that result in ‘similarity’ between observed samples, D, and simulated samples, D’, generated by θ. θ-values are sampled from the prior distribution π(θ). Furthermore, to reduce computational complexity, summary statistics S = (S_1_,S_2_,…,S_M_) are used to represent the key features of the original data—in other words, ƒ(θ|S) is used to approximate ƒ(θ|D). The ABC version of rejection sampling is then as follows:

For i = 1 to *N*

Sample parameters θ’ from the prior distribution π(θ)

Simulate data D’ using the tumor growth model described earlier with the sampled parameters θ’, and summarize D’ as S’.

Accept θ’ if d(S’, S) < ε, for a given threshold ε, where d(S’, S) is a measure of distance (which can be thought of as 1/‘similarity’) between S’ and S.

Adding extra non-informative or less informative summary statistics increases the noise in the measure of distance, and thereby increases the error of matching S’ to S [24,25]. Therefore, we must carefully select a minimal set of summary statistics that capture all important information regarding tumor growth. A number of methods have been invented to choose a concise set of summary statistics, ensuring that they maintain informativeness with regard to inferring posterior distributions for model parameters [25–29]. We explore related methods below and choose a set of summary statistics that performs well when estimating our model parameters. As is common, we define d(S’, S) as a variant of the Euclidean distance metric. Specifically, we use a variant of traditional Euclidean distance in which each statistic is weighted. Some summary statistics s* might be more informative for a particular parameter θ* than others, therefore, a higher weight on s* will help infer the posterior distribution of θ* [25,30]. So including the weights of each summary statistic, the distance metric is defined as:

$$\text{d}\left(\text{S}’,\text{S}\right)={\left|\left|\left(\text{S}’-\text{S}\right){\text{W}}^{\text{T}}\right|\right|}_{2}.$$ (2)

#### Summary statistics

Assume that *G* glands are sampled from the tumor. Each gland has *K*_*g*_ (1*≤g≤G*) mutations. The allele frequency of k-th mutation in g-th gland is denoted as *F*_*gk*_. In a diploid system, if a mutation is present in every cell of a gland, i.e. it has an allele frequency of 0.5, it is called a “fixed” mutation. Otherwise, the mutation is called non-fixed. We also define a “gland-specific” mutation as a mutation that is only present in one gland, while a “shared” mutation is one that is found in more than one gland.

Our analysis then uses the following summary statistics:

- The mean of the allele frequencies of all non-fixed mutations,
- The variance of the allele frequencies of all non-fixed mutation,
- The number of gland-specific mutations among non-fixed mutations,
- The number of gland-specific mutations among fixed mutations,
- The number of shared mutations among non-fixed mutations,
- The number of shared mutation among fixed mutations,
- The variance of the number of non-fixed mutation across all glands,
- The variance of the number of fixed mutation,
- The variance of the allele frequency of non-fixed mutations across the two tumor halves.

#### Selecting weights for summary statistics

In this section, we compare three methods to assign weights to summary statistics. As a baseline comparison we employ an analysis that uses equal weight for every statistic, and infers the parameters jointly. To reduce the dimensionality of the simulation study, which is already large and extremely computationally intensive, our perspective here will be to focus on one parameter of interest at a time. As a consequence, we will also learn which of our statistics are most informative for each of our parameters. We show results for two methods in which we infer parameters one-at-a-time (i.e. we infer marginal parameter posterior distributions). Thus, the second weighting method we use, the ‘local linear regression method’, applies local regression for the parameter of interest θ at each data point, based on each statistic, and uses the coefficient between parameter and statistic as the weight of that statistic (for details, see below). The third method we compare, ‘global linear regression’, proceeds similarly, but now utilizes a global measure of correspondence between parameter and each summary statistic to determine the weight for each statistic (again, we use the correlation coefficient).

#### Local linear regression method

Here we describe our second analysis method, that is based on local linear regression [31]. We conduct *N* simulations, each of which simulates a single dataset, and in each of which the *L* parameters were sampled from the prior distributions in Table 1. We denote the summary statistics observed in these *N* simulations by S**’** = (***s***_*1*_,***s***_*2*_,***s***_*3*_,*…*,***s***_*M*_), and denote the *L* generating parameter values by ϴ**’** = (***θ***_*1*_, ***θ***_*2*_, ***θ***_*3*_,*…*,***θ***_*L*_), where ***s***_*m*_ (1*≤m≤M*) and **θ**_*l*_ (1*≤l≤L*)are column vectors of length *N*. For a given parameter θ_*l*_, we wish to assess how much information each summary statistic carries regarding that parameter. In order to do this, we denote the collection of generating parameter values, and the resulting summary statistic values, by the pair (***θ***_*l*_, ***s***_*m*_). For each summary statistic *s*_*m*_, we then perform a linear regression of ***θ***_*l*_, on ***s***_*m*_ in the vicinity of *s*_*m*_ using the simulated datasets. We define this vicinity as the 100*η percent of simulated data points that are closest to *s*_*m*_ (in terms of their resulting summary statistic values, using the Euclidean distance metric), for some *k*. We denote these closest points by S’_m(1)_, S’_m(2)_,…, S’_m(*kN*)_ (where the subscript () denotes the rank ordered values, starting with the value closest to *s*_*m*_) and the corresponding generating parameters are denoted by ϴ’_*l*(1)_, ϴ’_l(2)_,…, ϴ’_l(*kN*)_. We then fit a linear regression using S’_m()_ as predictor variable and ϴ’_*l*()_ as response variable. The R-square measure of fit of the linear regression is recorded as *R*^*2*^_*lm*_. After all weights are calculated between the *l*^*th*^ parameter and *m*^*th*^ summary statistic, we define final normalized weights to use as:

$$W_{lm}=\frac{R_{lm}^{2}}{\sum_{l=1}^{L}R_{lm}^{2}}.$$ (3)

We use the above weights as the weights for the summary statistics in a subsequent ABC analysis for the parameter of interest, using previously unseen data.

#### Global linear regression method

The global correlation between a parameter and a summary statistic also indicates how informative that summary statistic is for a parameter. While it may be less accurate, in principle, locally, it will also be less subject to the noise that might arise from using just local values to estimate correlation. For that reason, our third method proceeds as above but now calculates statistic weights based on the R-square measure of fit, $R_{lm}^{2}$, between parameter *θ*_*l*_ and summary statistic *S*_*m*_ derived from a global linear regression for a set of simulated data. Again, these weights are then used when estimating that same parameter in previously unseen data in order to assess estimation performance of this weighting method. The weights, *W*_*lm*_, are defined and normalized in the same way as in Eq (3).

### Data simulation

#### Perfect synthetic data

When we generate simulated tumor data for our study we use the tumor growth model described earlier in this section. In our first set of analyses we explore a situation in which we have ‘complete’ data, in the sense that no noise (e.g. sampling variation) was simulated. For example, we assume that we can calculate the exact allele frequency of a mutation from the output of the simulation. In reality, of course, such data is subject to error, but the performance of our methods on this ‘perfect’ data provides a benchmark for the rest of our study.

#### Simulated data with variable read depth

While it is useful to benchmark the analysis method in this best case scenario, we also wish to assess how it performs in a realistic setting. In idealized models, the sequencing depth follows a Poisson-like distribution with a small variance [32,33]. However, in reality, the distribution of sequencing depth has a larger variance than would be predicted by such a model. Therefore, here, as is common, we use the more flexible negative binomial distribution, *X~NB(p*,*t)*, to model sequencing depth. If we think of the negative binomial distribution as the number of successes, *k*, in a sequence of independent Bernoulli trials (with success rate *p*) before the *t*^*th*^ failure, then,

$$f\left(k;t,p\right)=Pr\left(X=k\right)=\left(\begin{array}{c} k+t-1\\ k \end{array}\right)p^{k}{\left(1-p\right)}^{t},$$ (4)

for *k* = 1, 2, 3…. Based on this definition, the mean (*m*) and the variance (*v*) of the negative binomial distribution are:

$$m=\frac{pt}{1-p},$$ (5)

$$v=\frac{pt}{{\left(1-p\right)}^{2}}.$$ (6)

We can re-write these constraints as:

$$p=1-\frac{m}{v},$$ (7)

$$t=\frac{mv}{v-m}-m.$$ (8)

Given an average sequencing depth (*m*) with variance (*v*), we model the sequencing depth individually for each segregating locus based on the negative binomial distribution described above. For a given locus, we draw a number *k* from the negative binomial distribution with parameter *p* and *t* that are calculated by Eqs (7) and (8) to represent the sequencing depth at this locus. Having done this we use a binomial distribution *B(k*, *q)* with *q* equal to the true allele frequency of mutated (alternative) allele, to generate the number, *n*_*a*_ of reads sampled for the mutated (alternative) allele, with the remaining *k—n*_*a*_ reads being of the reference allele.

As discussed earlier, mutant alleles with frequency 0.5 in the simulated diploid data are referred to as “fixed”. However, when we are modeling the sequencing process itself, the exact allele frequency is no longer obtained. Instead we observe counts of the number of reads at any given position with each of the two possible allelic types. For this reason, at each locus we conduct a hypothesis test to determine whether or not we might regard the allele as “fixed”. Specifically, since sequencing depths are generated according to a binomial distribution, we use a standard binomial hypothesis test to assess whether we can reject the null hypothesis that the underlying *q*, the true allele frequency of mutated allele, is 0.5. If we do not reject the null hypothesis for a given mutation, then we consider this mutation as (potentially) fixed. Here type II errors will increase the number of mutations called as fixed, typically because when the actual allele frequency is relatively close to 0.5 the test will typically fail to reject the null hypothesis even though the mutation is actually not fixed. However, we reflect this same calling method in the production of simulated data during the ABC analysis of our synthetic data. Thus, while our anti-conservative calling of fixed sites can be expected to introduce some loss of precision in our analysis, there is no reason, a priori, to expect it to introduce bias for the estimation of the parameters.

#### Somatic mutation calling

To identify somatic mutations, raw genomic sequence reads were mapped to the 1000 Genomes (b37) build of the human genome reference with BWA (version 0.7.5a) using default settings [34]. The resulting alignments were processed using the GATK (version 2.8–1) base quality score recalibration, indel realignment, and duplicate removal (picardtools, version 1.103) following the GATK Best Practices recommendations [35–37]. Somatic single nucleotide variants were identified using MuTect version 1.1.4 [38]. MuTect was run using default parameters in the High Confidence (HC) mode along with dbSNP (version 137) and COSMIC (version 67) databases.

## Results

### Synthetic tumor data

#### Weight summary statistics

**Fig 2:**
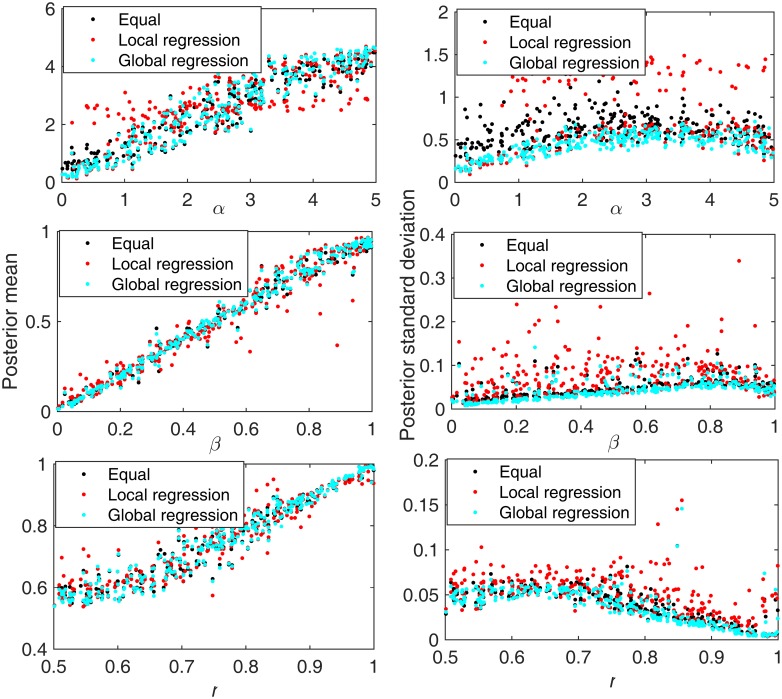
Summary of posterior distributions for each parameter under different weighting schemes. Black represents the results using equally weighted summary statistics. Red shows the results of using the R-square (coefficient of determination) of the local linear regression as the weight. Green corresponds to the results using weights given by the global linear regression. 300 simulated test tumors were included in this analysis. The x-axis is the true generating parameter value for each tumor. The top row shows results for mutation rate before gland formation. The middle row shows results for the mutation rate after gland formation, while the third row shows the results for the asymmetric division rate. The first column shows the mean of the posterior parameter estimate, while the second column shows the standard deviation of the posterior distribution.

Fig 2 shows the results of a simulation study comparing the performance of these three methods in estimating model parameters for our tumor growth model. For each simulated dataset we modeled tumor growth and then sampled 6 glands from each half of the resulting tumor. The other generating parameter values were: NCSC = 32, T3 = 100. The figures in the first column illustrate the mean of the posterior distributions for mutation rate before gland formation (top), mutation rate after gland formation (middle) and the asymmetric division rate (bottom), for 300 simulated test tumors, while the ones in the second column represent the posterior standard deviation. Each of the three methods generates good estimates, although there are some exceptional cases in which the posterior means deviated from the true values by large amounts when the local regression was used. This illustrates the noisier nature of estimates of local (as compared to global) regression between parameters and summary statistics. However, the three methods have differing performances in terms of the standard deviation of the posterior distribution (Fig 2, right column). The standard deviations are very high for the local regression method, again likely because local estimates of regression parameters are relatively unstable. The method that uses equal weights performs relatively well compared to the local regression methods. However, the posterior distributions generated by weighting the summary statistics by R-square measure of fit of the global linear regression have a consistent tendency to have the smallest standard deviations, indicating that the R-square measure of fit of the global linear regression serves as a better weighting method than other two methods for our analysis.

#### Minimum detectable allele frequency

A key feature of our simulated data is the allele frequency of each mutation. If a mutation occurs early during tumor development, its allele frequency will typically be large. For example, a mutation introduced to the tumor in the early stem cell divisions has allele frequency 0.5 in a diploid setting. If a mutation first appears in the constant growth phase (T3), the allele frequency can stay very low or can increase to 0.5, the relative probabilities of these outcomes depending on the asymmetric division rate and the number of generations that occur before the tumor is extracted. With real experimental data, because of the realities of sequencing technologies, we are not able to reliably detect mutations with very low allele frequencies, because they cannot be distinguished from sequencing errors. The ability to detect mutations with low allele frequency depends on the depth of the sequence data that has been collected for that tumor. For example, if the sequencing depth is 20, the lowest allele frequency that is detectable is 1/20, which is 0.05.

**Fig 3:**
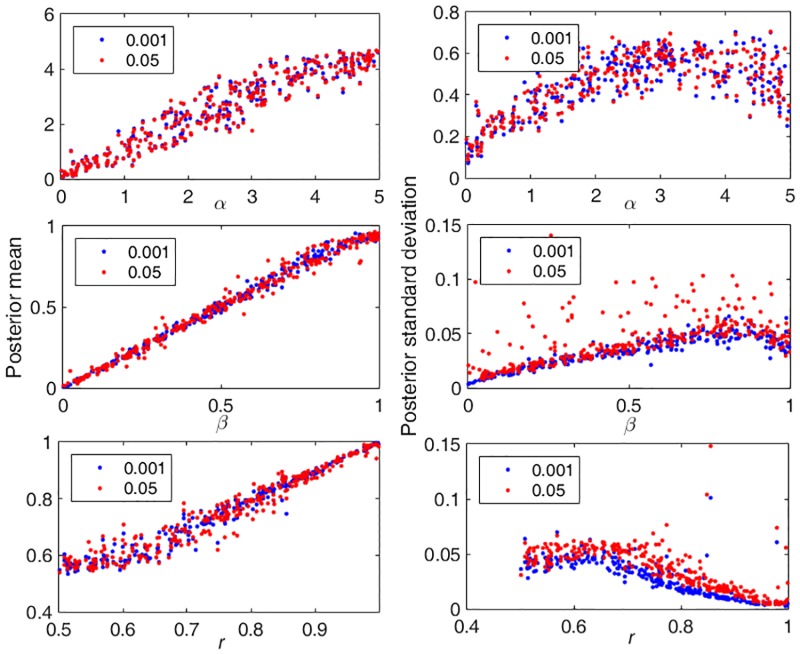
Different minimum detectable allele frequency. 300 tumors with a range of generating parameters were tested. Blue dots are for a threshold of 0.001 and red dots show results for a threshold of 0.05. 6 glands were sampled from each half. Other generating parameter values are: NCSC = 32, T3 = 100.

To explore this, we generated 300 test tumors, for each of a range of parameter values, using a range of lowest detectable allele frequency thresholds (to reflect the inability to reliably detect low frequency mutations). Mutations with frequency lower than this threshold were removed from the analysis. Fig 3 shows results for two such thresholds: 0.05 and 0.001. We show the means and standard deviations of the posterior distributions of the corresponding parameter estimates. As we can see, the minimum detectable allele frequency does not affect the estimation of the mutation rate before gland formation. Mutations before gland formation all become fixed, with allele frequency 0.5, due to the gland fission processes, and so are easily detected using either threshold. However, for the other two parameters, the detection threshold does have an impact on the posterior distributions. The means of the posterior distributions are largely unaffected, and are still centered around the true (generating) parameter values, but the variance increases with the higher threshold. This suggests that our ABC analysis continues to function well in this more realistic setting. However, we do see an effect in the standard deviations of the posterior distribution. These grow larger as the threshold value increases, reflecting the loss of data results from our lack of ability to detect lower frequency mutations.

#### Number of sampled glands

**Fig 4:**
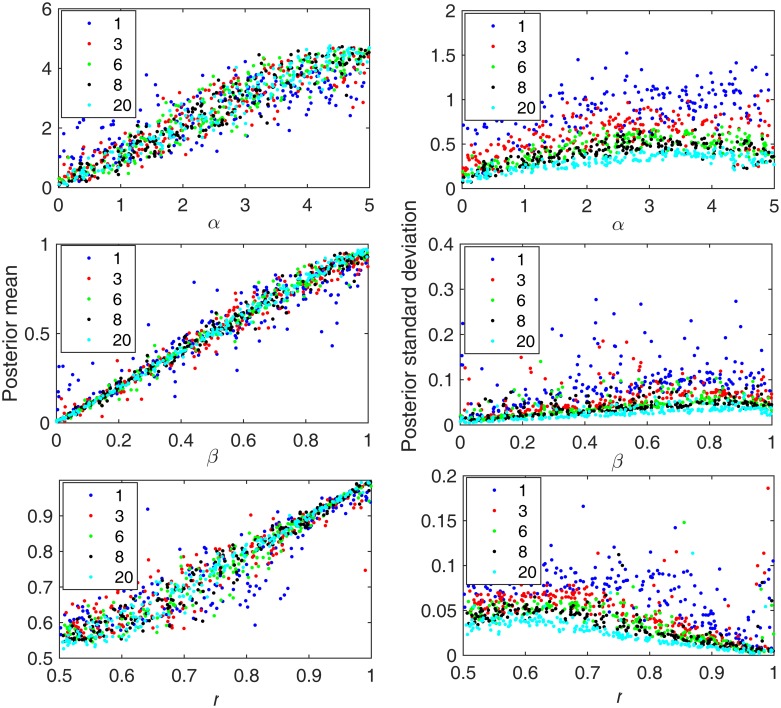
Summary of posterior distributions as a function of the number of glands sampled from each tumor (x-axis). Colors correspond to the different numbers of sampled glands (see key). Top row: mutation rate before gland formation; middle row: mutation rate after gland formation; Bottom row: asymmetric division rate. Other generating parameter values are: NCSC = 32, T3 = 100. The numbers in the legend represent the number of glands that were sampled from each half of the simulated tumors. The first column and second column show the mean and variance, respectively, of each parameter’s posterior distribution across 300 simulated tumors.

Another important question is how the number of glands we sample from a tumor affects our ability to estimate the model parameters. Intuitively, the more glands we sample the more information we will have about the tumor, and so a large number of sampled glands is of course preferred. However, the ability to sample glands from a tumor may be limited for practical reasons. Therefore, we explore how estimation performance trades off against number of glands sampled. To do this, we simulated tumor growth and harvested differing numbers of glands upon which to base the analysis. 300 such tumors were analyzed and posterior distributions of each parameter for each tumor were summarized via the mean and the standard deviation. As we can see in Fig 4, with only one gland sampled from each tumor half the posterior distributions have significantly larger standard deviations and the means of the posterior distributions deviate more from the true values. Among the three parameters tested, this is most severe for estimation of the mutation rate before gland formation. If we have only one gland sampled from each half, the summary statistics contain relatively little information about events very early during the tumorigenesis. More precisely, the heterogeneity within each tumor half is not captured by a single gland at all. However, with 3 or more glands, performance is relatively good (and improves as the number of sampled glands increases, as expected). We suggest that sampling 6 glands from each tumor half is a reasonable compromise. This number generates reasonably accurate estimates that have a small standard deviation. Therefore, we assume 6 glands from each tumor half in later analyses in this paper.

#### Adding experimental noise in the form of sampling variation

In the previous sections, we assumed that we had perfect data in the sense that all information is recorded completely and accurately. For example, the allele frequency of each mutation in the glands is assumed known, when detectable. We now add further, a more realistic filter to the data. For example, estimation of allele frequency will depend upon the quality of the sequencing data collected, which itself is subject to the sequencing technology used, the DNA quality, and requested sequencing depth, etc. As such, we obtain estimates of these underlying true data characteristics (for example, we may completely fail to detect a mutant allele, or estimate its frequency incorrectly). Also, some regions of the genome are hard to sequence, resulting in lower coverage in those regions. To explore these considerations we now examine the effect of sequencing depth, a key determinant of data quality, on the performance of our estimation. Higher coverage is always better from the perspective of data accuracy. But, of course, data is not free, so we explore what depth of coverage is necessary to ensure high quality estimation.

**Fig 5:**
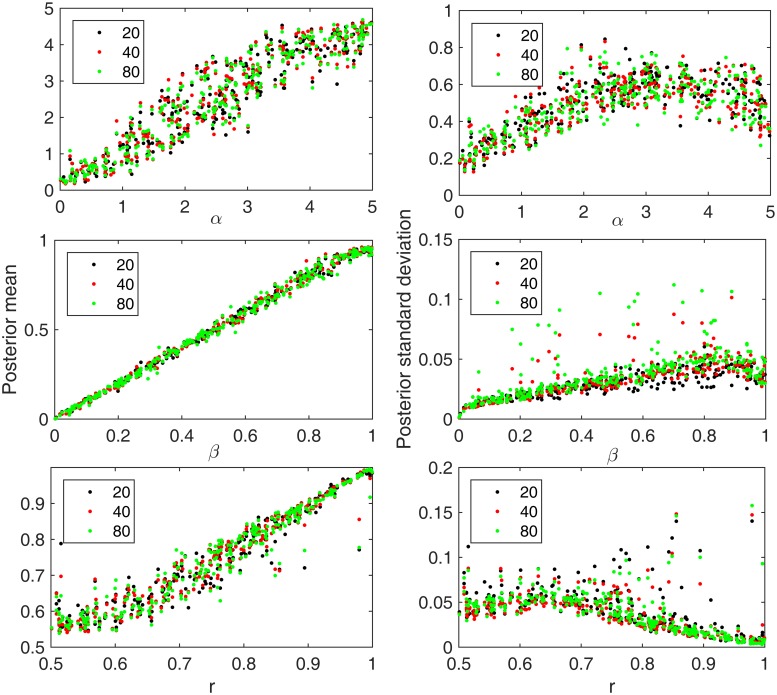
Summary of posterior distributions for different sequencing depths. The first column and second column show the mean and standard deviation of each parameter’s posterior distribution for the 300 simulated tumors respectively. The top row is the summary of for mutation rate before gland formation. The middle row is for the mutation rate after gland formation while the third row shows the results for the asymmetric division rate. The black dots, red dots and green dots represent the results when the mean sequencing depths are 20, 40, and 80 respectively, per single gland. We see little improvement of parameter estimation with even higher sequencing depths. The other generating parameter values used were: NCSC = 32, T3 = 100, and *v* = 1.1*m* for the negative binomial distribution used to generate sequencing depth.

Intuitively, we expect that higher sequencing depth should produce better results in parameter estimation. The real question is “how much (coverage) is enough?” As shown in Fig 5, a mean sequencing depth of just 20 appears to be sufficient to obtain good performance in our parameter estimates. Even with significantly higher mean sequencing depth, e.g. 80, the performance of the posterior distribution does not differ by much. There is no detectable difference between the posterior variances that result when using a depth of just 20. This demonstrates the advantage of the approximate Bayesian computation approach (or indeed any other model-based approach): performance can be robust to the presence of sampling error *so long as the processes resulting in that sampling error are captured in the model itself*, as they were here.

### Experimental data

Having shown that our parameter estimation procedure performs well in realistic settings, we close with an analysis of a small dataset. We focus on additional new *single-gland* exome sequencing data from one tumor first reported by Sottoriva et al., 2015 (tumor U). In this new experiment, sequence data was obtained for just one gland per half. While any conclusions drawn will clearly be tentative, the results earlier in this paper show that some power for parameter estimation remains. This data has been uploaded as supplementary data (“S1 File” and “S2 File”) for this paper.

#### Is there a mutation burst?

Sottoriva et al. proposed a big bang tumor growth model in which after transformation, some colorectal tumors develop as a single terminal expansion containing subclonal events in the absence of stringent selection [6]. Mutations occurring prior to transformation will be present in all cells of the tumor and by definition clonal, whereas after transformation only early arising mutations, will reach detectable frequencies in the tumors. One fundamental question arises: is there a mutation burst at the very beginning of the tumor development? In other words, is the mutation rate before the first gland formation much bigger than the mutation rate after the first gland formation? This question is key for understanding the early evolution of a tumor.

**Fig 6:**
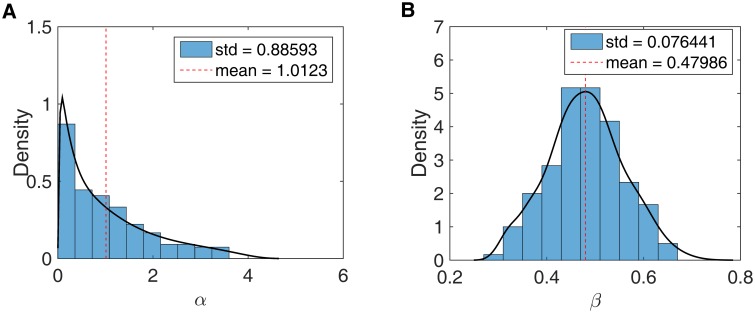
Posterior distributions of mutation rate for tumor U. (A) mutation rate before gland formation. (B) mutation rate after gland formation. The dashed line indicates the mean of the posterior distribution.

**Fig 7:**
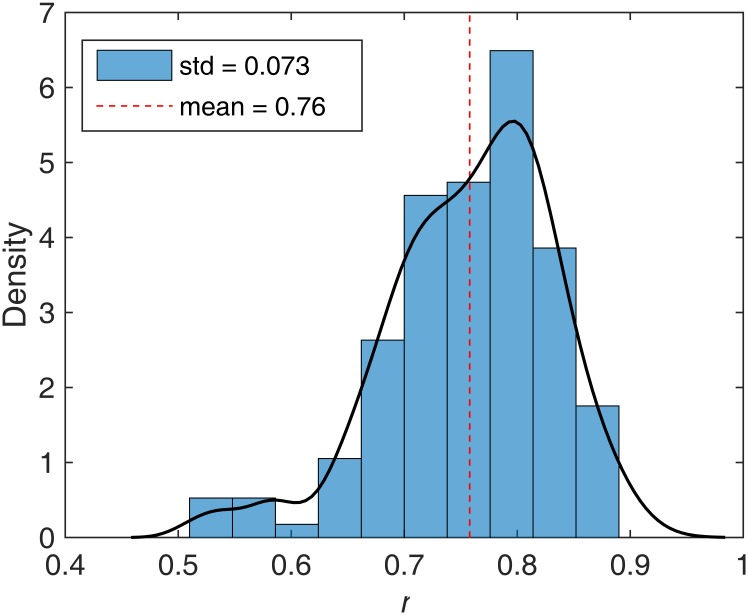
Posterior distribution of asymmetric division rate (x-axis) for tumor U. Dashed line indicates the mean of the posterior distribution.

Our results for analysis of tumor U are shown in Figs 6 and 7. Tumor U contains many copy number variations in the genome. We use the method described in Kang et al., 2015 to call integer copy numbers for each chromosomal segment. The allele frequency of each mutation then is adjusted according to the copy number of its location. (For example, if a mutation has frequency 0.33 in a region for which the copy number is 3, the mutation is interpreted as “fixed”.) We see that while the posterior distribution has relatively high variance, there is still signal in the data regarding mutation rate. The mean and the mode for mutation rate after gland formation are 0.48 and 0.5 respectively (Fig 6B), while the mutation rate before gland formation has mean 1.01 and mode 0.33. Based on this one tumor, no clear conclusion regarding the relative magnitudes of the two mutation rates can be drawn. While the posterior for the pre-gland mutation rates supports higher values, and that for the post-gland mutation rate does not, the posteriors have a large degree of overlap.

#### How stem cells divide?

The results of our simulation study on synthetic tumors suggest that we can successfully infer the asymmetric division rate. Applying the same procedure to the data from tumor U we obtain the results shown in Fig 7. We see that the mean of the posterior distribution is around 0.76 with little support for values around 0.5, at which point division would be random (50% symmetric and 50% asymmetric). Thus, even with data for just one tumor, and one gland from each side of that tumor, we see that CSCs in this tumor (at least) almost certainly undergo asymmetric division.

## Discussion

Inference regarding properties of tumor growth may well be crucial in understanding both their behavior and, ultimately, how best to impact growth through medical treatment. But tumor growth is non-trivial to understand because it is typically not observed. However, modern technologies allow high-resolution data to be collected. Here we focus on our ability to now collect data regarding sequence level mutation on small numbers of cells within a tumor gland (e.g. 10,000 cells). While this application of the technology is relatively new we have access to little actual data, our simulation study shows that model-based analyses based upon ABC have the ability to successfully infer key parameters of tumor growth using such data.

The number of mutations thought to have originated during the first several cell divisions, which can be detected by comparing the mutations profile in glands from multiregional samples, does not match the number generated by the normal mutation rate (10^−10^ to 10^−9^ per base per cell division). A mutation burst at early stage tumor growth has been proposed to explain this phenomenon [8]. However, no obvious evidence has been presented to rule out the possibility that the mutation rate in the tumor is elevated throughout development, rather than just during an initial ‘burst’. In this paper we demonstrated that the mutation rates both during the initial stage of tumor development and at the later stage can be estimated using sequence-level data study, even if such data is limited. Although the data from the sole single-gland data available to us, (from tumor U), were consistent with the idea of a burst, the posterior distributions of mutation rates in tumor U do not allow a decisive conclusion to be drawn (see Fig 6). However as more data, for more glands, is collected in future, our analysis framework is likely to allow investigators to decisively conclude whether or not such a burst has occurred.

Researchers have used biomarkers to confirm the existence of ‘stem cell like’ cells in various tumors [39,40]. However, the details of CSC behavior have been hard to uncover, in part because that behavior is hard to directly observe. As a consequence, the details of how CSCs divide are also unknown. Some experiments have shown that not all CSCs divide in the same manner. Some go through symmetric division and others undergo asymmetric division [41,42]. However, it is still unknown what determines how a CSC divides and with what probability CSCs utilize asymmetric division to produce progeny. Despite all of these unknowns, CSCs are thought to be a frequent cause of recurrence after treatment and have been targeted for therapeutics in many studies [43–45]. In this paper we demonstrated a method for estimating how CSCs divide in a given tumor. The posterior distribution of the asymmetric division rate probabilistically represents the behavior of a CSC. The model is constrained to assume equal symmetric division rates which yields a constant population size that fits our data. We could instead simulate additional variation in these rates so that the means are constant. This would add further sampling variability but not bias, suggesting even larger data sets are needed for precise inference. This is a topic of future research.

In previous work, we showed how to determine the number of CSC in each gland of a colorectal tumor[46]. Together, these results might be used to find correlations with the severity and resilience of a tumor, which might then be used to guide the individualized therapeutics.

We also show that our analysis performs robustly in the face of experimental noise when we consider both the limit of detectable allele frequency and variation of sequencing depth. This is because in a simulation-based method it is relatively straightforward to model the processes that result in such experimental noise. When ABC is performed, the perturbation caused by the noise in the data is also present in simulated data and therefore it is captured by the ABC procedure (Figs 3 and 5). We also show that some conclusions can be drawn even if we have just a single gland from each side of a single tumor (Figs 4, 6 and 7).

We focus on marginal analysis of parameters in this paper, modeling a situation in which there is a particular parameter that is of interest to the investigator. The method extends naturally to joint analysis of multiple parameters. However, for the experimental data presented in this paper, (one gland from each side of a single tumor), such a joint analysis would probably have little discriminative power.

In summary, the simulation study presented in this paper showed that the mutation rate at different stages of tumor development and the asymmetric division rate of CSCs can be retrieved based on mutation data collected from single gland sequencing. We also showed that relatively little data is required to extract at least some useful information regarding the existence of a mutation burst and the asymmetric division rate. Our ABC framework provides a widely-applicable tool for extracting information from genomic data, and in particular the parameters that govern the development of a tumor, which may potentially shed light on the post-diagnosis and post-surgery treatment.

## Supporting information

S1 File This file contains the somatic single nucleotide variants that were identified using MuTect (version 1.1.4). UA contains the information for one half of tumor U. MuTect was run using default parameters in the High Confidence (HC) mode along with dbSNP (version 137) and COSMIC (version 67) databases. A detailed explanation of each column in this file can be found here: http://archive.broadinstitute.org/cancer/cga/mutect_run.
(KEEP)

S2 File This file contains the somatic single nucleotide variants that were identified using MuTect (version 1.1.4). UB contains the information for the other half of tumor U. MuTect was run using default parameters in the High Confidence (HC) mode along with dbSNP (version 137) and COSMIC (version 67) databases. A detailed explanation of each column in this file can be found here: http://archive.broadinstitute.org/cancer/cga/mutect_run.
(KEEP)
